# Validation and *in vivo* characterization of research antibodies for Moesin, CD44, Midkine, and sFRP-1.

**DOI:** 10.12688/f1000research.138354.2

**Published:** 2024-04-23

**Authors:** Suzanne Doolen, Riham Ayoubi, Carl Laflamme, Ranjita Betarbet, Elizabeth Zoeller, Sean-Paul G. Williams, Haian Fu, Allan I. Levey, S. J. Sukoff Rizzo

**Affiliations:** 1 Aging Institute, University of Pittsburgh, Pittsburgh, Pennsylvania, 15219, USA; 2 Neurology and Neurosurgery, McGill University, Montreal, Québec, H3A 2B4, Canada; 3 Center for Neurodegenerative Disease, Emory University, Atlanta, Georgia, 30322, USA; 4 Department of Pharmacology, Emory University, Atlanta, Georgia, 30322, USA; 5 Chemical Biology Discovery Center, Emory University, Atlanta, Georgia, 30322, USA; 6 Winship Cancer Institute, Emory University, Atlanta, Georgia, 30322, USA; 7 Neurology, Emory University, Atlanta, Georgia, 30322, USA; 8 Goizueta Alzheimer's Disease Research Center, Emory University, Atlanta, Georgia, USA

**Keywords:** Alzheimer’s disease, Moesin, sFRP-1, Midkine, antibody characterization, Western blot, mice, 5xFAD

## Abstract

**Background:**

A major goal of the Target Enablement to Accelerate Therapy Development for Alzheimer’s disease (TREAT-AD) program is to develop and identify high-quality tools to test target or mechanistic hypotheses. As part of this initiative, it is important that commercial reagents including research antibodies being used to interrogate drug targets have confirmed validation data in knock-out cell lines. Ideally, these antibodies should also have utility for both *in vitro* and *in vivo* studies such that the levels of target proteins in target tissues can be quantified.

**Methods:**

We evaluated commercial antibodies against TREAT-AD protein targets Moesin (Uniprot ID: P26038), CD44 (Uniprot ID: P16070), Midkine (Uniprot ID: P21741) and Secreted frizzled-related protein 1, referred to as “sFRP-1” (sFRP-1; Uniprot ID: Q8N474). Moesin, Midkine and sFRP-1, that were confirmed as selective based on data in knock-out cell lines. Western blot analysis was used to compare protein levels in brain homogenates from a mouse model with AD-relevant pathology (5XFAD) versus age-matched C57BL/6J control mice.

**Results:**

Anti-Moesin ab52490 reacted in mouse brain homogenate with a predicted molecular weight of 68 kDa. Moesin protein expression was 2.8 times higher in 5xFAD compared to WT. Anti-CD44 ab189524 reacted with a band at the predicted size of 82 kDa. CD44 protein expression was 1.9 times higher in 5xFAD compared to WT. Anti-Midkine AF7769 reacted with a band ~16 kDa and a 17.8 times greater expression in 5xFAD compared to WT. Anti-sFRP-1 ab267466 reacted with a band at 35 kDa as predicted. sFRP-1 protein expression was 11.9 times greater in 5xFAD compared to WT.

**Conclusions:**

These data confirm the utility of these selective commercially available antibodies against Moesin, CD44, Midkine, and sFRP-1 for *in vivo* studies in mice and provide insight into the use of 5XFAD mice for *in vivo* target engagement studies for these target proteins.

## Introduction

Alzheimer’s Disease (AD) is a debilitating neurodegenerative disorder affecting an estimated 6.7 million Americans age 65 years and older with no clear understanding of the disease mechanism ( ‘2023 Alzheimer’s disease facts and figures’ 2023). Leading mechanistic hypotheses, such as the amyloid and tau hypotheses, have yet to generate fully efficacious therapies that can prevent or stop AD. As part of a national effort to develop therapeutics and biomarkers for AD, the Accelerated Medicines Partnership for Alzheimer’s Disease (AMP-AD) Consortium has been leveraging unbiased molecular profiling data at the genomic, transcriptomic, proteomic and metabolomic levels to further understanding of AD pathogenesis. Recent studies as part of AMP-AD have used a tandem mass tag mass spectrometry (TMT-MS) approach of postmortem brain tissues to reveal new AD-related protein co-expression modules ( Seyfried *et al*. 2017; Johnson *et al*. 2020). These protein modules were strongly correlated with higher neuropathological burden and worse cognitive outcomes ( Seyfried *et al*. 2017; Johnson *et al*. 2020). One such module referred to as Module 4 or “M4” included Moesin and CD44 and had the strongest correlation with higher neuropathological burden and worse cognitive outcomes ( Seyfried *et al*. 2017; Johnson *et al*. 2020). M4 was enriched in microglial and astrocytic proteins and contains proteins involved in response to inflammation. Another novel AD-associated module, M42 or the matrisome module, had a significant correlation with global pathology and is a source of promising therapeutic targets and biomarkers for AD. M42, which was not present in RNA networks, contained several proteins that have previously been identified by TMT-MS and shown to be correlated with amyloid-beta deposition in brain as well as with Midkine and sFRP-1 ( Higginbotham *et al*. 2020; Ping *et al*. 2018; Bai *et al*. 2020; Johnson *et al*. 2018, 2022).

Although the quality of research antibodies has been long been an issue ( Weller 2018), technical, economic and sociological challenges obstacles impede proper antibody characterization. The recent democratization of the CRISPR-Cas9 technology allows the generation of ideal isogenic controls for proper antibody characterization ( Laflamme *et al*. 2019, 2021). We have characterized commercial antibodies against Moesin ( Alshafie *et al*. 2023), Midkine ( Ayoubi *et al*. 2023a), sFRP-1 ( Ayoubi *et al*. 2023b). As commercial research antibodies become available and validated in commercially available human knock-out cell lines, it is essential to extend the characterization of these antibodies in murine tissues to enable their use in AD-mouse models.

The 5xFAD mouse is a well-characterized transgenic model that manifests Aβ plaque deposition as early as 4-6 months of age ( Jawhar *et al*. 2012; Oakley *et al*. 2006). Here we have characterized the expression of the TREAT-AD target proteins Moesin, CD44, Midkine and sFRP-1 in the 5xFAD mouse model in comparison to age-matched non-transgenic C57BL/6J controls. This allows not only for confirmation of the presence and/or changes in the protein level of target proteins but these antibodies also serve as tools for future *in vivo* target engagement studies as we evaluate the potential of novel therapeutic agents to modulate disease.

## Methods

In vivo studies were in line with NIH Guide for The Care and Use of Laboratory Animals ( Council 2011) and followed the Animal Research: Reporting of *In Vivo* Experiments (ARRIVE) guidelines ( Kilkenny *et al*. 2010; Doolen & Rizzo 2023b). Prior to study initiation (April 2023) all in vivo procedures were reviewed approved by the University of Pittsburgh Institutional Animal Care and Use Committee (IACUC). All efforts were made to ameliorate harm to the animals by adherence to the 3 Rs alternatives; replacement, reduction and refinement ( Tannenbaum and Bennett 2015).

Adult male and female 5xFAD (JAX MMRRC Stock #034840; B6.Cg-Tg (APPSwFlLon,PSEN1*M146L*L286V)6799Vas/Mmjax) and C57BL/6J (JAX stock #000664) were used for these studies. These 5XFAD mice are congenic on the C57BL/6J substrain and were received directly from the Jackson laboratory which maintains a genetic stability and specific pathogen-free biosecurity program, and provides appropriate assurances and genotyping protocols ( www.jax.org). Subjects were 7-12 weeks upon arrival to the University of Pittsburgh animal facility and were group-housed within sex (up to 4 per cage) in a dedicated mouse housing room with a 12hr:12hr light:dark cycle (lights on at 7:00am). The University of Pittsburgh animal facility is a specific pathogen-free facility that is fully accredited by the American Association for Accreditation of Laboratory Animal Care (AAALAC). An Animal Welfare Assurance is on file with OPRR-NIH. Subjects were maintained in the dedicated mouse housing facility with *ad libitum* food and water until the intended age of the experiments which was pre-determined based on our previous published characterization of the 5XFAD mouse model ( Oblak *et al*. 2021). Specifically, 5XFAD mice and controls were aged to 6-9 months which is an age in which 5XFAD mice demonstrate significant AD relevant pathophysiology including amyloid plaque deposition in brain and neuroinflammation, relative to C57BL/6J controls ( Oblak *et al*. 2021) Tissue samples were collected during the light cycle. (All efforts were undertaken to ameliorate animal suffering including appropriate methods of anaesthesia for terminal tissue collection.

The total number of animals used for all experiments were n= 4 5XFAD and n=4 C57BL/6J. These studies were initially conducted with minimal sample sizes (n=3-4) in line with historical published data for 5XFAD mice showing robust phenotypes of AD-related pathologies ( Oblak *et al*. 2021) and in line with protocols from the MODEL-AD consortium ( ADKnowledgeportal.synapse.org).

Western blots were reproduced and confirmed from initial pilot data, under blinded conditions. The experimenter was not aware of the group allocation during the experiment and data analysis. A staff member who serves the role of a colony manager that was not involved directly with the experiment coded the samples as A and B. The staff member provided the unblind code after the data were analyzed by the experimenter and reviewed for quality control by the supervisor. No samples were excluded from this study.

### Mouse brain collection

For terminal tissue collection, mice were anesthetized with isoflurane to the surgical plane of anesthesia, and brains were collected following decapitation. The brain was extracted and rinsed in ice-cold PBS, cerebellum was removed, and the cortex was bisected into left and right hemispheres then snap frozen and stored at -80 °C until use. Each hemibrain was weighed and then immersed in 1 ml/100 mg tissue homogenization buffer (THB; 2 mM Tris (pH 7.4), 250 mM sucrose 0.5 mM EDTA 0.5 mM EGTA) supplemented with 1X Pierce Protease Inhibitor (Thermo Scientific #A32953) and 1x Phosphatase Inhibitor Cocktail 2 (Sigma-Aldrich #P5726, St. Louis, MO). The tissue was homogenized for 20 sec on ice with a Benchmark D1000 hand-held homogenizer beginning at medium and increasing to high speed. Total protein concentration of the resulting homogenate was measured using a Bradford assay ( Bradford 1976). Briefly, 5 μl of homogenate diluted 10× in PBS was added to a 96-well plate. Pierce™ Coomassie Plus (Bradford) Assay Reagent (Life Technologies, Chicago, IL, USA; 250 μl) was added to each well and the plate was read at 595 nm using a SprectraMax i3x (Molecular devices) with SoftMax Pro V7.0.2. The protein concentration was calculated by comparing to a standard curve generated by adding varying known concentrations of Albumin Standard (0, 125, 250, 500, 750, 1000, 1500 and 2000 μl/ml; (Thermo Scientific #23210, Rockford, IL, USA) in duplicate to wells.

### Western blots

Cell cultures: Wild type (WT) and MDK knockout (KO) HAP1 cells were obtained from Horizon Discovery. Cell lines were prepared by first washing 3× with PBS then starved for ~18 hrs. Culture media were collected and centrifuged for 10 min at 500 × g to eliminate cells and larger contaminants, then for 10 min at 4500 × g to eliminate smaller contaminants. Culture media were initially concentrated using Amicon Ultra-15 Centrifugal Filter Units (MilliporeSigma cat. number UFC9010) by centrifuging at 4000 × g for 15 min. The resulting 500 μl of the concentrated media were centrifuged again at 4000 × g for 15 min using Amicon Ultra- 0.5 Centrifugal Filter Units (MilliporeSigma cat. number UFC5010) to 200 μl. ~30 μg of protein was loaded and processed for Western blot using the Midkine antibody (AF7769). Western blot was performed using a large 10-20% gradient polyacrylamide gel and transferred on a nitrocellulose membrane. Proteins on the blot were visualized with Ponceau staining which is scanned to show together with the Western blot. The blot was blocked with 5% milk for 1 hr, and the Midkine antibody (AF7769) was incubated overnight at 4°C with 5% bovine serum albumin in TBS with 0.1% Tween 20 (TBST). Following three washes with TBST, the peroxidase conjugated secondary antibody was incubated on the membrane at a dilution of ~0.2 μg/ml in TBST with 5% milk for 1 hr at room temperature followed by three more washes with TBST. The membrane was incubated with ECL from Pierce (cat. number 32106) prior to detection with HyBlot CL autoradiography films from Denville (cat. number 1159T41). The MDK KO cell line can be confirmed based on the results of the Western blot, as the Midkine signal appears in the lane loaded with the WT cells but disappears in lane loaded with the MDK KO cells. Predicted band size: ~16 kDa.

Brain Homogenates: Homogenate from N=2 male and n=2 female 9 month aged 5XFAD subjects and homogenate from N=4 age-matched individual male C57BL/6J subjects, which served as WT controls, were included on each gel. Equal amounts of protein (25 μg) samples were prepared in 1x loading buffer (4x: 4 ml 100% glycerol, 2.4 ml 1M Tris/HCl (pH 6.8), 0.8 g SDS, 4 mg bromophenol blue, 3.1 ml H2O, 0.5 ml beta-mercaptoethanol) and incubated at 95 °C for 5 min. Samples were separated with SDS-polyacrylamide gel electrophoresis (4–15% Mini-PROTEAN ^®^ TGX™ Precast Protein Gels, 10-well, 30 μl, Bio-rad #4561083) using 120 V for 60 min, then transferred onto a nitrocellulose membrane using a Trans-Blot SD Semi-Dry Electrophoretic transfer cell (Bio-rad #170-3940) at 25 V for 30 min. Non-specific binding was blocked using EveryBlot Blocking Buffer (Bio-rad #12010020) for 5 min at room temperature with gentle rocking. Primary antibodies were prepared in EveryBlot Blocking buffer at the dilutions indicated in Table 1. Blots were immersed in primary antibody solutions overnight at 4 °C with gentle rocking. Membranes were then washed with 1X TBST (3 × 5 min) and immersed in 1:1000 fluorescent secondary antibody (Moesin, CD44 and sFRP-1, StarBright blue 700 goat anti-rabbit IgG, Bio-rad #12004161; Midkine, IRDye 800CW Donkey anti-Goat IgG, Licor, Lincoln, NE, USA) and 1:2000 hFAB™ Rhodamine Anti-GAPDH Antibody (Bio-rad #12004167) for 1 h at room temperature. Membranes were then washed (2 × 5 min in 1X TBST; 2 × 5 min in 1X TBS) then scanned with a Bio-rad ChemiDoc MP Imaging System. Pixel intensity was quantified using ImageJ, the public domain NIH Image program (available at http://rsb.info.nih.gov/nih-image/). Statistical analyses and graphing were performed using an unpaired T-test GraphPad Prism Version 9.3.1 (San Diego, CA).

**Table 1.** Summary of commercial antibodies use in western blot assays.

Target protein	Product #	Company	Source	Dilution used	RRID (Antibody Registry)	Clonality	Clone #	Conc. (μg/μL)	Fold change in 5xFAD brain v C57BL/6J
Moesin	ab52490	Abcam	Rabbit	1:1000	AB_881245	recombinant-mono	EP1863Y	0.19	2.8
CD44	ab189524	Abcam	Rabbit	1:500	AB_2885107	recombinant-mono	EPR18668	0.47	1.9
Midkine	AF7769	R&D Systems	Sheep	1:200	AB_291796	polyclonal	-	1.0	17.8
sFRP-1	ab267466	Abcam	Rabbit	1:1000	AB_2904616	recombinant-mono	EPR23092-253	0.46	11.9

## Results

### Selection of antibodies

**Figure 1.**
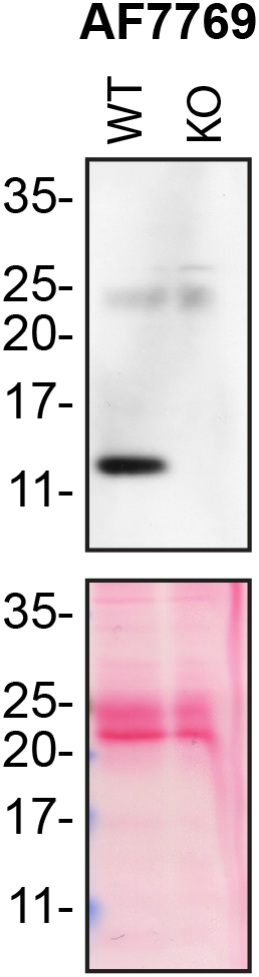
Midkine antibody screening by Western Blot on culture media. Lysates of HAP1 (WT and MDK KO) were prepared, and ~30 μg of protein was processed for Western blot with the Midkine antibody. The Ponceau stained transfers of each blot are presented to show equal loading of WT and KO media and protein transfer efficiency from the acrylamide gels to the nitrocellulose membrane.

**Figure 2.**
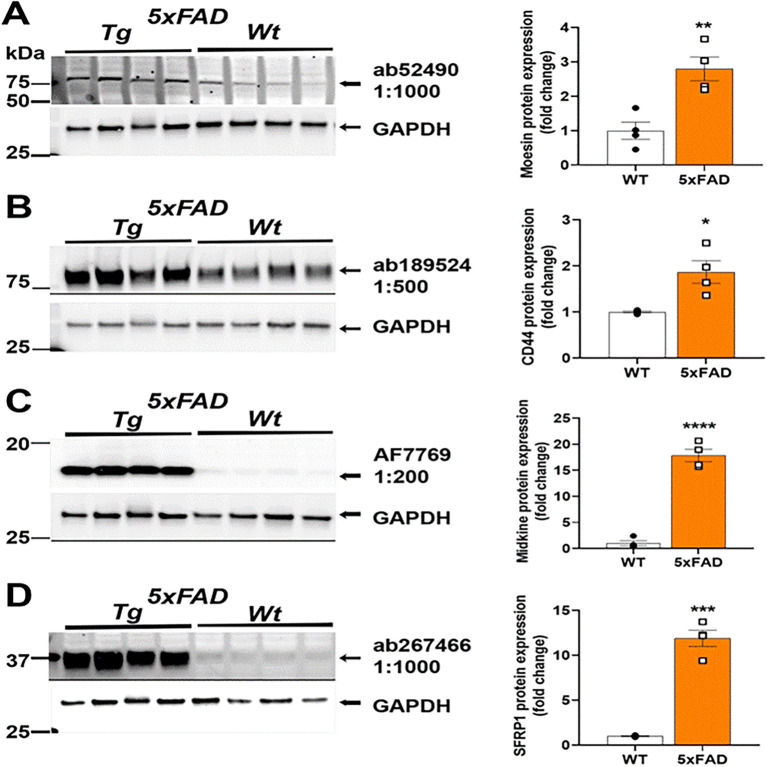
Analysis of Moesin, CD44, Midkine and sFRP-1 protein level in 5XFAD AD mouse model. Representative Western blots (left) and quantification (right). (A) Anti-Moesin; (Abcam #ab52490) reacted in mouse brain homogenate with a predicted molecular weight of 68 kDa and 2.8 times greater expression in 5XFAD relative to WT. (B) Anti-CD44 (Abcam #ab189524) reacted with a band at the predicted size of 82 kDa and 1.9 times greater expression in 5XFAD relative to WT. (C) Anti-Midkine (R&D Systems #AF7769) reacted with a band ~16 kDa with 17.8 times greater expression in 5XFAD relative to WT. (D) Anti-sFRP-1 (Abcam #ab267466) reacted with a band at 35 kDa as predicted with 11.9 times greater expression in 5xFAD relative to WT. Brightness and contrast were adjusted in figure for visualization purposes and were applied equally to entire blot. *P<0.05, **P<0.01, ***P<0.001, ****P<0.0001 by unpaired *t-test*.

We have characterized most commercially available antibodies against Moesin ( Alshafie *et al*. 2023), Midkine ( Ayoubi *et al*. 2023a), sFRP-1 ( Ayoubi *et al*. 2023b) and CD44 ( Ayoubi *et al*. 2021) using human WT and knockout cell lines for three research applications, including Western blot. For this study, we selected antibodies based on the following criteria: *i)* high antibody selectivity by WB based on our previous antibody characterization studies, *ii)* predicted reactivity with mouse based on the internal validation from the manufacturer, *iii)* band at the expected size by Western blot using mouse brain lysate ( Figure 2) and *iv)* antibody clonality as we prioritize renewable reagents such as mouse monoclonal or rabbit recombinant antibodies. We selected the recombinant anti-Moesin ab52490, the recombinant anti-CD44 ab189524 and the recombinant anti-sFRP-1 ab267466 antibodies as they met our criteria. However, none of the previously characterized Midkine antibodies was expected to react with mouse Midkine. By searching on antibody manufacturers’ website, we identified the polyclonal antibody AF7769 from Bio-techne generated to specifically detect mouse Midkine. We validated AF7769 as we have done previously ( Ayoubi *et al*. 2023a) ( Figure 1). AF7769 met our selection criteria and was further selected for the downstream experiments. The underlying data for this study has been uploaded to Zenodo ( Doolen & Rizzo 2023a).

### Protein expression is increased in mouse models of AD

We next used the selected antibodies on protein extracts from cortex of male and female 9-month aged 5xFAD mouse brain homogenates and compared to age-matched C57BL/6J controls. Our immunoblotting results depicting their reactivity in mouse brain homogenate are shown in Figure 2. The antibody source information and the fold change in protein levels in 5xFAD are in Table 1. Anti-Moesin ab52490 reacted in mouse brain homogenate with a predicted molecular weight of 68 kDa and its expression was 2.8 times higher in 5xFAD compared to WT mouse brain ( Figure 2A). Anti-CD44 ab189524 reacted with a band at the predicted size of 82 kDa and its expression was 1.9 times higher in 5xFAD compared to WT mouse brain ( Figure 2B). Anti-Midkine AF7769 reacted with a band ~16 kDa and a 17.8 times greater expression in 5xFAD compared to WT mouse brain ( Figure 2C). Anti-sFRP-1 ab267466 reacted with a band at 35 kDa as predicted and its expression was 11.9 times greater in 5xFAD compared to WT mouse brain ( Figure 2D). Interestingly, both Midkine and sFRP-1 are secreted proteins and could still be identified in crude protein extracts from homogenates, prepared as described above. For analysis, an unpaired T-test was performed using Graphpad Prism Version 9.3.1 for Windows ( www.graphpad.com).

## Discussion

Validated reagents are important tools for studying underlying biological mechanisms that may contribute to disease and are a critical component of rigorously interrogating potential novel therapeutic targets for AD. Contrastingly, reagents such as antibodies that are not rigorously characterized could lead to spurious, irreproducible data and significant waste of resources. The present studies extend the characterization of antibodies that have been validated in knock-out cell lines to western blots to confirm their utility for *in vivo* studies. Given the evidence reporting that postmortem brain tissue from AD patients have elevated levels of Moesin, CD44, Midkine, and sFRP-1 relative to case controls, we selected an AD mouse model to evaluate these antibodies. As predicted, male and female 5XFAD mice at an age at which significant amyloid plaque deposition is present ( Oblak *et al*. 2021, 2022), demonstrated increases in Moesin, CD44, Midkine, and sFRPR1, relative to age- and sex- matched non-transgenic controls. These data confirm the utility of these specific antibodies as validated reagents for western blotting of brain tissue, and also highlight the presence of these target proteins in a mouse model of amyloidosis. While western blotting is only semi-quantitative and reflect changes in antibody signal, we believe these taken together, provide an approach for confirming and extending the validation of antibodies from *in vitro* tissue preparations to *in vivo* assays and also provide insight into the utility of the 5XFAD mouse model for studying Moesin, CD44, Midkine, and sFRP-1.

## Data Availability

Zenodo: in vivo characterization of research antibodies for Moesin, CD44, Midkine, and sFRP-1. https://doi.org/10.5281/zenodo.8043971 ( Doolen & Rizzo 2023a). Updated version: https://zenodo.org/doi/10.5281/zenodo.8043970. Zenodo: ARRIVE checklist for ‘Validation and in vivo characterization of research antibodies for Moesin, CD44, Midkine, and sFRP-1.’ https://doi.org/10.5281/zenodo.8160965 ( Doolen & Rizzo 2023b). Data are available under the terms of the Creative Commons Attribution 4.0 International license (CC-BY 4.0).

## References

[ref1] 2023 Alzheimer’s disease facts and figures: Alzheimers Dement. 2023. 10.1002/alz.13016 36918389

[ref2] Alshafie W Ayoubi R Fotouhi M : The identification of high-performing antibodies for Moesin for use in Western Blot, immunoprecipitation, and immunofluorescence. *F1000Res.* 2023;12. 10.12688/f1000research.130126.3 PMC10724652 38106655

[ref3] Ayoubi R Southern K Laflamme C : The identification of high-performing antibodies for Midkine for use in Western blot and immunoprecipitation. *F1000Res.* 2023a;12. 10.12688/f1000research.133899.3 PMC10755264 38161428

[ref4] Ayoubi R Southern K Laflamme C : The identification of high-performing antibodies for Secreted frizzled-related protein 1 (sFRP-1) for use in Western Blot and immunoprecipitation. *F1000Res.* 2023b;12. 10.12688/f1000research.133479.2 PMC11420615 39319244

[ref5] Ayoubi R Alshafie W McPherson PS : Antibody Characterization Report for CD44 antigen. 2021. 10.5281/zenodo.4730966

[ref6] Bai B Wang X Li Y : Deep Multilayer Brain Proteomics Identifies Molecular Networks in Alzheimer’s Disease Progression. *Neuron.* 2020;105:975–991.e7. 10.1016/j.neuron.2019.12.015 31926610 PMC7318843

[ref7] Bradford MM : A rapid and sensitive method for the quantitation of microgram quantities of protein utilizing the principle of protein-dye binding. *Anal. Biochem.* 1976;72:248–254. 10.1016/0003-2697(76)90527-3 942051

[ref8] Council, National Research: *Guide for the Care and Use of Laboratory Animals.* Eighth ed. Washington, DC: The National Academies Press;2011.

[ref9] Doolen S Rizzo SJS : in vivo characterization of research antibodies for Moesin, CD44, Midkine, and sFRP-1.[Dataset]. *Zenodo.* 2023a. 10.5281/zenodo.8043971 PMC11497330 39444645

[ref10] Doolen S Rizzo SJS : ARRIVE checklist Doolen 2023. *Zenodo.* 2023b. 10.5281/zenodo.8160965

[ref11] Higginbotham L Ping L Dammer EB : Integrated proteomics reveals brain-based cerebrospinal fluid biomarkers in asymptomatic and symptomatic Alzheimer’s disease. *Sci. Adv.* 2020;6. 10.1126/sciadv.aaz9360 33087358 PMC7577712

[ref12] Jawhar S Trawicka A Jenneckens C : Motor deficits, neuron loss, and reduced anxiety coinciding with axonal degeneration and intraneuronal Abeta aggregation in the 5XFAD mouse model of Alzheimer’s disease. *Neurobiol. Aging.* 2012;33(196):e29–e40. 10.1016/j.neurobiolaging.2010.05.027 20619937

[ref13] Johnson ECB Carter EK Dammer EB : Large-scale deep multi-layer analysis of Alzheimer’s disease brain reveals strong proteomic disease-related changes not observed at the RNA level. *Nat. Neurosci.* 2022;25:213–225. 10.1038/s41593-021-00999-y 35115731 PMC8825285

[ref14] Johnson ECB Dammer EB Duong DM : Large-scale proteomic analysis of Alzheimer’s disease brain and cerebrospinal fluid reveals early changes in energy metabolism associated with microglia and astrocyte activation. *Nat. Med.* 2020;26:769–780. 10.1038/s41591-020-0815-6 32284590 PMC7405761

[ref15] Johnson ECB Dammer EB Duong DM : Deep proteomic network analysis of Alzheimer’s disease brain reveals alterations in RNA binding proteins and RNA splicing associated with disease. *Mol. Neurodegener.* 2018;13:52. 10.1186/s13024-018-0282-4 30286791 PMC6172707

[ref16] Kilkenny C Browne WJ Cuthill IC : Improving bioscience research reporting: the ARRIVE guidelines for reporting animal research. *PLoS Biol.* 2010;8:e1000412. 10.1371/journal.pbio.1000412 20613859 PMC2893951

[ref17] Laflamme C Edwards AM Bandrowski AE : Opinion: Independent third-party entities as a model for validation of commercial antibodies. *New Biotechnol.* 2021;65:1–8. 10.1016/j.nbt.2021.07.001 34246180 PMC12955693

[ref18] Laflamme C McKeever PM Kumar R : Implementation of an antibody characterization procedure and application to the major ALS/FTD disease gene C9ORF72. *elife.* 2019;8. 10.7554/eLife.48363 31612854 PMC6794092

[ref19] Oakley H Cole SL Logan S : Intraneuronal beta-amyloid aggregates, neurodegeneration, and neuron loss in transgenic mice with five familial Alzheimer’s disease mutations: potential factors in amyloid plaque formation. *J. Neurosci.* 2006;26:10129–10140. 10.1523/JNEUROSCI.1202-06.2006 17021169 PMC6674618

[ref20] Oblak AL Cope ZA Quinney SK : Prophylactic evaluation of verubecestat on disease- and symptom-modifying effects in 5XFAD mice. *Alzheimers Dement (N Y).* 2022;8:e12317. 35846156 10.1002/trc2.12317 PMC9281365

[ref21] Oblak AL Lin PB Kotredes KP : Comprehensive Evaluation of the 5XFAD Mouse Model for Preclinical Testing Applications: A MODEL-AD Study. *Front. Aging Neurosci.* 2021;13:713726. 10.3389/fnagi.2021.713726 34366832 PMC8346252

[ref22] Ping L Duong DM Yin L : Global quantitative analysis of the human brain proteome in Alzheimer’s and Parkinson’s Disease. *Sci. Data.* 2018;5:180036. 10.1038/sdata.2018.36 29533394 PMC5848788

[ref23] Seyfried NT Dammer EB Swarup V : A Multi-network Approach Identifies Protein-Specific Co-expression in Asymptomatic and Symptomatic Alzheimer’s Disease. *Cell Syst.* 2017;4:60–72.e4. 10.1016/j.cels.2016.11.006 27989508 PMC5269514

[ref24] Tannenbaum J Bennett BT : Russell and Burch’s 3Rs then and now: the need for clarity in definition and purpose. *J. Am. Assoc. Lab. Anim. Sci.* 2015;54:120–132. 25836957 PMC4382615

[ref25] Weller MG : Ten Basic Rules of Antibody Validation. *Anal. Chem. Insights.* 2018;13:1177390118757462. 29467569 10.1177/1177390118757462 PMC5813849

