# Supplementary material for: Validation and in vivo characterization of research antibodies for Moesin, CD44, Midkine, and sFRP-1
Source: F1000Res. 2024 Apr 23;12:1070. Originally published 2023 Aug 31. [Version 2] doi: 10.12688/f1000research.138354.2 (PMC11497330; doi:10.12688/f1000research.138354.2)
Supplement: Peer review history

# Reviewer report: Reviewer response for version 2

**DOI:** 10.5256/f1000research.164518.r326999

**Published:** 2024-10-21

**Authors:** Steve Jean

**Affiliations:** 1 Université de Sherbrooke,, Sherbrooke, QC, Canada

In this study, the authors assessed validated antibodies against Moesin, CD44, and sFRP-1 on brain extracts from wild-type or the Alzheimer disease 5xFAD mouse model for their ability to detect changes in their respective target protein levels. Additionally, they demonstrate the specificity of a Midkine antibody using WT and Midkine KO HAP1 cells and show its usefulness on mouse brain extracts.

 Altogether, this straightforward study demonstrates the efficacy of four independent antibodies. The original data is provided along with a detailed methodology. In their revised version, the authors have appropriately addressed comments from the previous reviewers. I only have minor points to address.

1. Correct the typo in the first line of the second paragraph: ‘ *Although the quality of research antibodies has been long been an issue…’ *
2. Based on the methodology, the western blot provided in Figure 1 is from the concentrated culture media. Please change ‘lysates’ to ‘culture media’ on the first lane, as a lysate would correspond to the lysed cells.
3. The ab52490 western blot is ‘reflected’ horizontally. This is apparent as the ‘wave’ in the gel is opposite between moesin and GAPDH WBs and easily observed when compared to the original data presented in the unblinded Excel file. Please correct the WB.
4. The targets of the four antibodies tested could be written in panels A to D of Figure 2 to ease data interpretation.
5. The methods should better describe the methodology used to quantify the WB. The Excel file shows normalization to GAPDH first, followed by the ratio calculation to WT extracts. This could be easily spelled out in the methodology.

Is the work clearly and accurately presented and does it cite the current literature?

Yes

If applicable, is the statistical analysis and its interpretation appropriate?

Yes

Are all the source data underlying the results available to ensure full reproducibility?

Yes

Is the study design appropriate and is the work technically sound?

Yes

Are the conclusions drawn adequately supported by the results?

Yes

Are sufficient details of methods and analysis provided to allow replication by others?

Partly

Reviewer Expertise:

Membrane trafficking, Drosophila melanogaster, proteomics, genetics, RAB GTPases, phosphoinositides.

I confirm that I have read this submission and believe that I have an appropriate level of expertise to confirm that it is of an acceptable scientific standard.

# Reviewer report: Reviewer response for version 2

**DOI:** 10.5256/f1000research.164518.r268997

**Published:** 2024-04-29

**Authors:** Sarah C Hopp

**Affiliations:** 1 University of Texas Health Science Center San Antonio, San Antonio, Texas, USA

The authors addressed my previous concerns.

Is the work clearly and accurately presented and does it cite the current literature?

Yes

If applicable, is the statistical analysis and its interpretation appropriate?

Yes

Are all the source data underlying the results available to ensure full reproducibility?

Yes

Is the study design appropriate and is the work technically sound?

Yes

Are the conclusions drawn adequately supported by the results?

Yes

Are sufficient details of methods and analysis provided to allow replication by others?

Partly

Reviewer Expertise:

Microglia in Alzheimer's disease

I confirm that I have read this submission and believe that I have an appropriate level of expertise to confirm that it is of an acceptable scientific standard.

# Reviewer report: Reviewer response for version 1

**DOI:** 10.5256/f1000research.151544.r249991

**Published:** 2024-03-22

**Authors:** Michelle Erickson

**Affiliations:** 1 University of Washington, Seattle, Washington, USA

This article evaluates specific antibodies for proteins upregulated in 5xFAD mouse brain, as found by Western blotting.  Results are straightforward and potentially useful to the reserach community.  Specific comments are below:

 1. A human knockout cell line is used to validate antibody specificity.  As this study specifically focuses on mice, it would be important to understand whether specificity is also observed in mouse knockout cells.

 2.  5xFAD mice can show sex-specific differences in AD-associated pathological changes.  Group allocation of males and females should be clarified.

 3.  Western blotting done here is only semi-quantitative. The increases reflect changes in antibody signal rather than protein expression.

 4.  The underlying data in Zenodo aren't appropriately labeled.  It's unclear what blot goes with what antibody or what the lanes are.  Also, the blots appear to have 12 lanes whereas only 8 are shown in the figure.  What are the other 4 lanes? 

 5.  Showing the full blots and clearly indicating what is thought to be the specific band is important, and ideally should be included in the main body of the manuscript.

Is the work clearly and accurately presented and does it cite the current literature?

Partly

If applicable, is the statistical analysis and its interpretation appropriate?

Partly

Are all the source data underlying the results available to ensure full reproducibility?

Partly

Is the study design appropriate and is the work technically sound?

Partly

Are the conclusions drawn adequately supported by the results?

Partly

Are sufficient details of methods and analysis provided to allow replication by others?

Partly

Reviewer Expertise:

Blood-brain barrier, Alzheimer's disease, aging, inflammation, neurotoxicology, pharmacology

I confirm that I have read this submission and believe that I have an appropriate level of expertise to confirm that it is of an acceptable scientific standard, however I have significant reservations, as outlined above.

# Response

**Published:** 2024-04-06

**Authors:** Suzanne Doolen

**Affiliations:** Aging, University of Pittsburgh, Pittsburgh, Pennsylvania, USA

This article evaluates specific antibodies for proteins upregulated in 5xFAD mouse brain, as found by Western blotting.  Results are straightforward and potentially useful to the research community.  Specific comments are below:

1. A human knockout cell line is used to validate antibody specificity.  As this study specifically focuses on mice, it would be important to understand whether specificity is also observed in mouse knockout cells.

**RESPONSE: We acknowledge the importance of ensuring specificity and cross-  reactivity, especially when working with antibodies across species. To address this concern, we conducted Western blot (WB) experiments using human knockout (KO)  cell lines and identified several specific antibodies in WB for all four proteins. The antibodies selected in this study were those predicted by the manufacturers to bind to  the corresponding murine proteins. We further confirmed specificity in WB by  comparing the band sizes between human and murine proteins. It's important to note that for all four proteins, the human and murine proteins are expected to exhibit very  similar molecular weights.**

             2.  5xFAD mice can show sex-specific differences in AD-associated pathological changes. Group allocation of males and females should be clarified.

**RESPONSE: We have now clarified that both male and female 5XFAD were used (n=2 per sex) for these studies in several places including methods and results.**

1. Western blotting done here is only semi-quantitative. The increases reflect changes in antibody signal rather than protein expression.

**RESPONSE: Thank you for this important point. We have added this to the discussion.**

  

1. The underlying data in Zenodo aren't appropriately labeled.  It's unclear what blot goes with what antibody or what the lanes are.  Also, the blots appear to have 12 lanes whereas only 8 are shown in the figure.  What are the other 4 lanes?

**RESPONSE: We have now updated the Zenodo notebook and added a file https://zenodo.org/search?q=parent.id%3A8043970&f=allversions%3Atrue&l=list&p=1&s=10&sort=version that shows the** **additional lanes were other animal models in our lab as we  were surveying other strains in addition to 5XFAD for these experiments and wanted   to maximize all lanes on the gel that were not other 5XFAD mice. Specifically, this was the SAA mouse model (JAX strain# 034711) which had not yet been fully characterized (Lanes 10-13) and Lanes 14 and 15 were n=2 19 month aged WT mice. We chose to only include 5XFAD in the publication given their extensive  characterization and their age-matched WT controls. Since the SAA mouse model did   not have full characterization data yet (e.g. amyloid and/or tau, etc.) and we only had  n=2 19-month aged WT mice and no 19-month aged 5XFAD to compare to we did not  include these in the publication.**

  

1. Showing the full blots and clearly indicating what is thought to be the specific band is important, and ideally should be included in the main body of the manuscript.

**RESPONSE: This has now been updated in the Zenodo notebook** **https://zenodo.org/search?q=parent.id%3A8043970&f=allversions%3Atrue&l=list&p=1&s=10&sort=version” that shows the specific bands which is in addition to what is shown in Figure 2.**

# Reviewer report: Reviewer response for version 1

**DOI:** 10.5256/f1000research.151544.r236011

**Published:** 2024-02-15

**Authors:** Sarah C Hopp, Caitlyn Fastenau

**Affiliations:** 1 University of Texas Health Science Center San Antonio, San Antonio, Texas, USA; 2 University of Texas Health Science Center San Antonio, SAN ANTONIO, TX, USA

The work is clear and concise with consideration of the previous work that highlight the significance of validating the antibodies of interest.

 It would be helpful to have more information on the functions of the four different proteins targeted by the different antibodies, and what the changes in expression in the 5XFAD brain lysate might mean biologically. 

1. The authors should be commended for their clear and reproducible methods with appropriate levels of detail, particularly the methods for selecting the antibodies. 
2. However, some details from the methods were missing:
3. Which sex of 5XFAD and WT mice were used for the Western blotting experiment in Figure 2? There are noted sex differences in pathology between sexes, so knowing which mice are males and females could be helpful.
4. The methods for Figure 1 are missing. Were the cell lysates and Western blots prepared in the same way as the brain lysate? What were the methods for the Ponceau? How was MDK KO confirmed in the HAP1 cells? Where were the HAP1 cells obtained from? How were they grown?
5. What t-test was run on the data in Figure 2?

Is the work clearly and accurately presented and does it cite the current literature?

Yes

If applicable, is the statistical analysis and its interpretation appropriate?

Yes

Are all the source data underlying the results available to ensure full reproducibility?

Yes

Is the study design appropriate and is the work technically sound?

Yes

Are the conclusions drawn adequately supported by the results?

Yes

Are sufficient details of methods and analysis provided to allow replication by others?

Partly

Reviewer Expertise:

Microglia in Alzheimer's disease

We confirm that we have read this submission and believe that we have an appropriate level of expertise to confirm that it is of an acceptable scientific standard, however we have significant reservations, as outlined above.

# Response

**Published:** 2024-04-06

**Authors:** Suzanne Doolen

**Affiliations:** Aging, University of Pittsburgh, Pittsburgh, Pennsylvania, USA

The work is clear and concise with consideration of the previous work that highlight the significance of validating the antibodies of interest.

 It would be helpful to have more information on the functions of the four different proteins targeted by the different antibodies, and what the changes in expression in the 5XFAD brain lysate might mean biologically. 

1. The authors should be commended for their clear and reproducible methods with appropriate levels of detail, particularly the methods for selecting the antibodies. 

**RESPONSE: Thank you to the reviewers for this acknowledgement.**

1. However, some details from the methods were missing:

**RESPONSE: we appreciate the feedback and have added points for clarity as suggested.**

1. Which sex of 5XFAD and WT mice were used for the Western blotting experiment in Figure 2? There are noted sex differences in pathology between sexes, so knowing which mice are males and females could be helpful.

 
**RESPONSE: As described in the second paragraph in the methods section both male and female 5XFAD mice were used for these studies. For clarity we have now revised the western blots methods to state: “Homogenate from N=2 male and N=2 female 9-month aged 5XFAD subjects and homogenate from N=4 age-matched individual male C57BL/6J subjects”. We have also added this detail in the results section. There were no differences at this age between male and female 5XFAD mice for these proteins and although only male WT were used as the age-match control (due to females being used as breeders), based on previous studies in 9 month aged WT mice there was no effect of sex in these proteins.**

1. The methods for Figure 1 are missing. Were the cell lysates and Western blots prepared in the same way as the brain lysate? What were the methods for the Ponceau? How was MDK KO confirmed in the HAP1 cells? Where were the HAP1 cells obtained from? How were they grown?

**RESPONSE: The cell lysates and brain homogenates had different preparations for the western blots. The details for the cell culture westerns have now been added as follows:**

**Wild type (WT) and *MDK* knockout (KO) HAP1 cells were obtained from Horizon Discovery.  Cell lines were prepared by first washing 3× with PBS then starved for ~18 hrs. Culture media were collected and centrifuged for 10 min at 500 × g to eliminate cells and larger contaminants, then for 10 min at 4500 × g to eliminate smaller contaminants.** **Culture media were initially concentrated using Amicon Ultra-15 Centrifugal Filter Units (MilliporeSigma cat. number UFC9010) by centrifuging at 4000 × g for 15 min. The resulting 500 μl of the concentrated media were centrifuged again at 4000 × g for 15 min using Amicon Ultra- 0.5 Centrifugal Filter Units (MilliporeSigma cat. number UFC5010) to 200 μl.** **~30μg of protein was loaded and processed for Western blot using the Midkine antibody (AF7769). Western blot was performed using a large 10-20% gradient polyacrylamide gel and transferred on a nitrocellulose membrane. Proteins on the blot were visualized with Ponceau staining which is scanned to show together with the Western blot. The blot was blocked with 5% milk for 1 hr, and the Midkine antibody (AF7769) was incubated overnight at 4°C with 5% bovine serum albumin in TBS with 0.1% Tween 20 (TBST). Following three washes with TBST, the peroxidase conjugated secondary antibody was incubated on the membrane at a dilution of ~0.2 μg/ml in TBST with 5% milk for 1 hr at room temperature followed by three more washes with TBST. The membrane was incubated with ECL from Pierce (cat. number 32106) prior to detection with HyBlot CL autoradiography films from Denville (cat. number 1159T41).** **The *MDK *KO cell line can be confirmed based on the results of the Western blot, as the Midkine signal appears in the lane loaded with the WT cells but disappears in lane loaded with the *MDK* KO cells. Predicted band size: ~16 kDa.**

1. What t-test was run on the data in Figure 2?

**RESPONSE: An unpaired t-test was performed using Graphpad Prism Software Version 9.3.1 for Windows. These details have now been added in the figure legend and the methods.**
